# Cardiac adipose tissue volume assessed by computed tomography is a specific and independent predictor of early mortality and critical illness in COVID-19 in type 2-diabetic patients

**DOI:** 10.1186/s12933-022-01722-2

**Published:** 2022-12-31

**Authors:** Etienne Charpentier, Alban Redheuil, Olivier Bourron, Samia Boussouar, Olivier Lucidarme, Mohamed Zarai, Nadjia Kachenoura, Khaoula Bouazizi, Joe-Elie Salem, Guillaume Hekimian, Matthieu Kerneis, Zahir Amoura, Yves Allenbach, Stephane Hatem, Anne-Caroline Jeannin, Fabrizio Andreelli, Franck Phan, COVID-19 APHP. SU Group, Romain Tilmont, Romain Chenu, Louise Meyfroit, Nicoletta Pasi, Schahrazed Larbi-Messaoud, Suzanne Laroche, Cécile Ciangura, Marc Popelier, Sophie Jacqueminet, Marine Halbron, Agnès Hartemann

**Affiliations:** 1 Sorbonne Université, Unité d’imagerie cardiovasculaire et thoracique, Hôpital La Pitié Salpêtrière (AP-HP), Laboratoire d’Imagerie Biomédicale, INSERM, CNRS, Institute of Cardiometabolism and Nutrition, Paris, France, Paris, France; 2 Institute of Cardiometabolism and Nutrition ICAN, Paris, France; 3 Laboratoire d’Imagerie Biomédicale, INSERM, CNRS, Institute of Cardiometabolism and Nutrition, Sorbonne Université, Paris, France; 4 Sorbonne Université, Département de diabétologie, Hôpital La Pitié Salpêtrière (AP-HP), Institute of Cardiometabolism and Nutrition, Paris, France, Paris, France; 5 Centre de Recherche Des Cordeliers, INSERM, UMR_S 1138, Paris, France; 6 Service d’imagerie specialisee et d’urgence SISU, Hôpital Pitié Salpêtrière, Assistance Publique-Hôpitaux de Paris, Laboratoire d’Imagerie Biomédicale, INSERM, CNRS, Sorbonne Université, Paris, France; 7 Department of Pharmacology, CIC-1901, INSERM, Assistance Publique-Hôpitaux de Paris (APHP), Sorbonne Université, Paris, France; 8 Assistance Publique-Hôpitaux de Paris (AP-HP), Hôpital La Pitié-Salpêtrière, Service de Médecine Intensive Réanimation, Sorbonne Université, Paris, France; 9 AP-HP, Hôpital La Pitié-Salpêtrière, ACTION Study Group, Département de Cardiologie, Sorbonne Université, Paris, France; 10 Service de Médecine Interne 2, Centre National de Référence Maladies Systémiques Rares et Histiocytoses, Institut e3M, Hôpital de La Pitié-Salpêtrière, AP-HP, Sorbonne Université, 75013 Paris, France; 11 AP-HP, Département de Médecine Interne Et Immunologie Clinique, Hôpital Pitié-Salpêtrière, Sorbonne Université, Paris, France; 12 Nutrition and ObesitiesSystemic Approaches (NutriOmics) Research Unit, INSERM, UMRS U1269, Sorbonne Université, Paris, France

**Keywords:** Type 2 diabetes (T2D), Cardiac adipose tissue, Computed tomography, COVID-19, Mortality, Intensive care

## Abstract

**Background:**

Patients with type 2-diabetes mellitus (T2D), are characterized by visceral and ectopic adipose tissue expansion, leading to systemic chronic low-grade inflammation. As visceral adiposity is associated with severe COVID-19 irrespective of obesity, we aimed to evaluate and compare the predictive value for early intensive care or death of three fat depots (cardiac, visceral and subcutaneous) using computed tomography (CT) at admission for COVID-19 in consecutive patients with and without T2D.

**Methods:**

Two hundred and two patients admitted for COVID-19 were retrospectively included between February and June 2020 and distributed in two groups: T2D or non-diabetic controls. Chest CT with cardiac (CATi), visceral (VATi) and subcutaneous adipose tissue (SATi) volume measurements were performed at admission. The primary endpoint was a composite outcome criteria including death or ICU admission at day 21 after admission. Threshold values of adipose tissue components predicting adverse outcome were determined.

**Results:**

One hundred and eight controls [median age: 76(IQR:59–83), 61% male, median BMI: 24(22–27)] and ninety-four T2D patients [median age: 70(IQR:61–77), 70% male, median BMI: 27(24–31)], were enrolled in this study. At day 21 after admission, 42 patients (21%) had died from COVID-19, 48 (24%) required intensive care and 112 (55%) were admitted to a conventional care unit (CMU). In T2D, CATi was associated with early death or ICU independently from age, sex, BMI, dyslipidemia, CRP and coronary calcium (CAC). (p = 0.005). Concerning T2D patients, the cut-point for CATi was  > 100 mL/m^2^ with a sensitivity of 0.83 and a specificity of 0.50 (AUC = 0.67, p = 0.004) and an OR of 4.71 for early ICU admission or mortality (p = 0.002) in the fully adjusted model. Other adipose tissues SATi or VATi were not significantly associated with early adverse outcomes. In control patients, age and male sex (OR = 1.03, p = 0.04) were the only predictors of ICU or death.

**Conclusions:**

Cardiac adipose tissue volume measured in CT at admission was independently predictive of early intensive care or death in T2D patients with COVID-19 but not in non-diabetics. Such automated CT measurement could be used in routine in diabetic patients presenting with moderate to severe COVID-19 illness to optimize individual management and prevent critical evolution.

## Keypoints

- Prognostic value of pericardiac fat tissue assessed using CT to predict adverse outcomes for T2D patients admitted for COVID-19 infection
- A disctinct phenotypic pattern was found between diabetic patients and controls with respect to adverse outcom:. classicl risk factors such  as age or sex adetermine the prognosis in controls  whereas the metabolic and inflammatory profile determine outcome in diabetics.
- Fast and reliable measurement of cardiac adipose tissue using CT. The assessment of this parameter could be useful to better stratify individual risk in diabetic patients with COVID-19 and adapt patient management of patients in routine practice.

## Background

COVID-19, a disease related to Severe Acute Respiratory Syndrome Coronavirus 2 (SARS-CoV-2) has rapidly spread globally since December 2019. The number of affected patients still increases, saturating national healthcare systems worldwide with persistent mortality in high-risk patients. In this context, the precise evaluation of individual risk to optimize patient management and therapeutic strategies based on discriminant prognostic tools remains central. Several clinical, radiological and biological predictors of COVID-19-related mortality have been described in the last two years including obesity, diabetes, high blood pressure, lung disease severity on CT scan and serum biomarkers of inflammation [1–6]. In particular, COVID-19 severity is dramatically increased in diabetic patients [7]. Visceral adipose tissue expansion is known to be associated with metabolic severity in diabetes. We recently reported [8] that increased cardiac adipose tissue index (CATi), an ectopic adipose tissue, and plasmatic IL6 are significantly related to early mortality and ICU requirement in diabetic patients with COVID-19, irrespective of obesity and could suggest to consider early preventive anti-inflammatory therapies. Abdominal visceral fat expansion has also been related to poor outcome in COVID-19 [9] but without comparison to CATi. Furthermore, it remains unclear whether the relationship between adipose tissue expansion including CATi and outcome in the setting of COVID-19 is more specific to diabetic patients or should be of concern to non-diabetics. The specific role of diabetes may be one of the reasons for inter-study heterogeneity found across studies included by Liu K. et al. in a recent meta-analysis confirming the association between EAT measures and COVID-19 severity and outcomes [10] including results from Bihan H. et al. from a group of 100 COVID-19 patients of whom only 42 had diabetes [11]. We hereby evaluate and compare the predictive value for early ICU or death of three adipose tissues (cardiac, visceral and subcutaneous) at admission for COVID-19 infection in consecutive patients with and without type-2 diabetes mellitus (T2D).

## Methods

### Study population

We retrospectively included 202 patients admitted for COVID-19 (positive SARS-CoV-2 PCR) during the first epidemiological peak between February and June 2020 in a large tertiary care academic center. These patients were distributed in two groups: T2D or non-diabetic as controls. Control patients were matched for age and sex with diabetic patients. Blood analysis at admission included glycemia, leukocyte, polymorphonuclear neutrophils (PMNs), lymphocyte and platelet counts, C-reactive protein, troponin-T, fibrinogen, creatinine, glomerular filtration rate estimation, AST and ALT.

Multidetector chest computed tomography (CT) was performed at admission to evaluate COVID-19 pneumonia severity. Subsequently, admitted patients were either hospitalized in a conventional medical unit (CMU) or in an intensive care unit (ICU) as required according to clinical severity criteria.

This ancillary monocentric observational study was based on a COVID-19 cohort approved by the local ethics committee CER-SU 2020-14 and registered as NCT04320017 (ClinicalTrials.gov). According to local legislation all study participants could withdraw their participation in the study.

### Outcome data

All clinical and biological data was collected from digital hospital admission and follow-up records. Outcome was collected at day 21 from the centralized hospital data recording ICU admission and mortality. The outcome endpoint was a composite criteria including death or ICU admission at 21 days of hospital admission.

### CT acquisition protocol

All patients underwent non-ECG gated helicoidal thoracic acquisitions at 120 kV with 0.6 mm collimation on either a dual source SOMATOM Definition Flash or EDGE scanner (Siemens Healthineers, Erlangen, Germany). Acquisitions were performed with or without contrast media injection.

### CT image analysis

COVID-19 related lung involvement was measured semi-quantitatively and reported according to the parenchymal extension of lung lesions using a standardized visual scale including ground glass and/or condensation as: minimal (< 10%), moderate (10–25%), extensive (25–50%) and severe (> 50%).

Since acquisitions were non-ECG-gated, we used the validated CAC-DRS score to quantify coronary calcium burden [12] graded from 0 to 3 as follows: 0: very low calcium; 1: mildly increased calcium; 2: moderately increased calcium; 3: moderately to severely increased calcium.

**Fig. 1:**
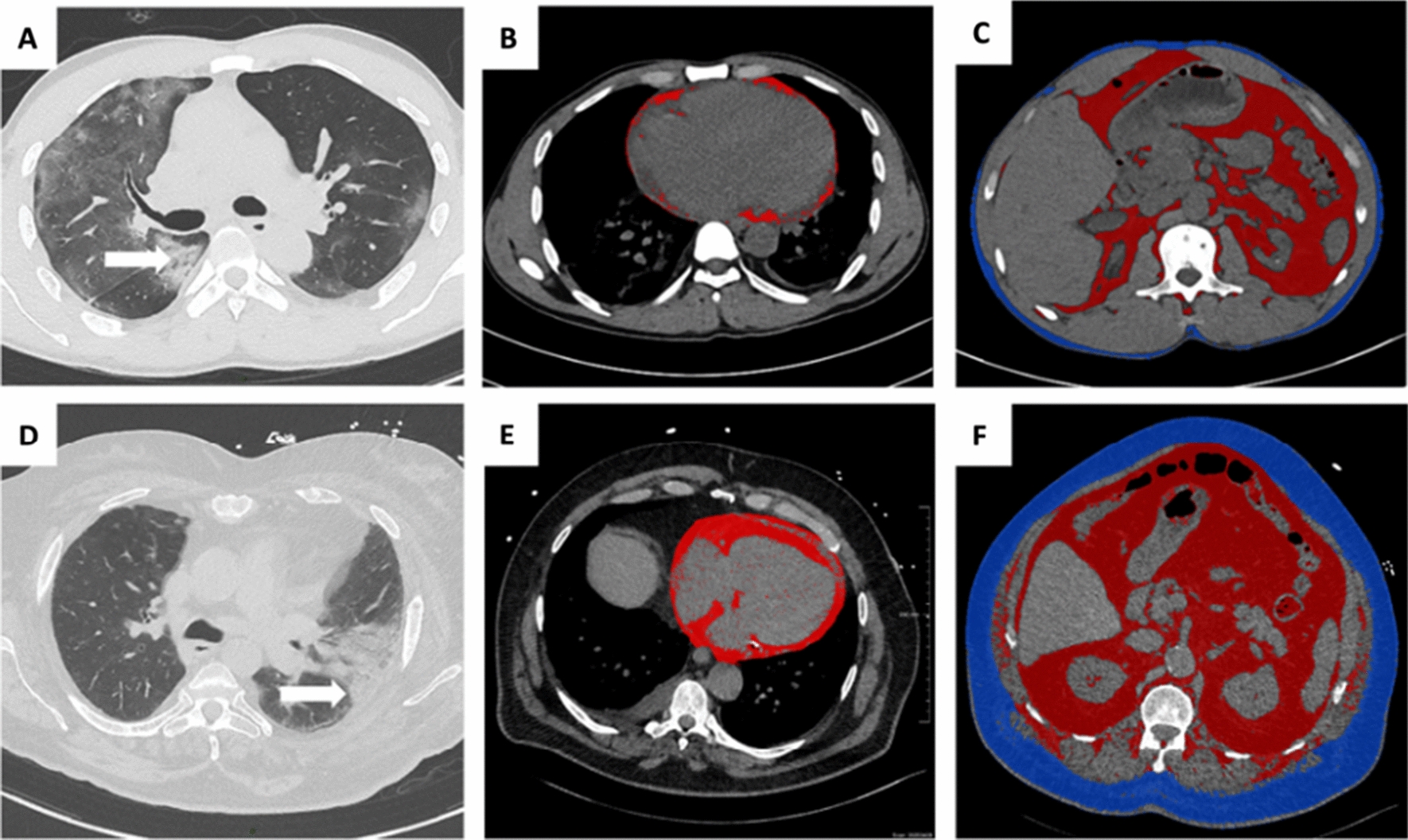
Illustration of CT imaging biomarkers assessed in two patients admitted for COVID-19 infection including chest CT for lung severity and cardiac, visceral and subcutaneous abdominal adipose tissue. Top row: A 44-year-old man from the control group presented extensive lung involvement with predominant ground-glass lesions and few consolidations with aeric bronchogram (**A**, arrows), with a low CATi score (**B**, red overlay), low VATi (**C**, red overlay) and low SATi (**C**, blue overlay). Bottom row: A 79-year-old man with type-2 diabetes shows moderately extensive lung involvement but with predominant consolidation lesions (**D**, arrows), with a high CATi score (**E**, red overlay), high VATi (**C**, red overlay) and high SATi (**C**, blue overlay). *CATi* cardiac adipose tissue indexed, *VATi* visceral adipose tissue indexed, *SATi* subcutaneous adipose tissue indexed (SATi)

We assessed adipose tissue imaging biomarkers related to different fat components using a semi-automated AI based segmentation method (Siemens Healthineers Frontier) including the following parameters (typical segmentation results are illustrated in Fig. 1):

- The total cardiac adipose tissue (CAT) in mL was measured after automated AI-based segmentation of the cardiac area with thresholding centered on the density range of adipose tissue values (-190 to -30 Hounsfield Units*).*
- The visceral abdominal fat (VAT) and the subcutaneous fat (SAT) were measured semi-automatically between L1 and L2 vertebral bodies. In this stack of images, draw external and internal contours of the abdominal wall were drawn, and an automated segmentation of visceral and subcutaneous fat was performed centered on the density range of adipose tissue values (-190 to -30 Hounsfield Units*)*.

These adipose tissue volumes were normalized to body surface area providing indexed parameters CATi, VATi and SATi respectively, in mL/m^2^.

The average time to perform thoracic and abdominal adipose fat tissue post processing was  < 10 min by patient including image data management and in the order of 10 s for computation. for CAT alone. In Fig. 1, we illustrate the CT imaging biomarkers in two patients with favorable and unfavorable courses.

### Statistical analysis

Numeric variables were reported as median and interquartile range (IQR) and qualitative variables as frequencies and percentages. To compare differences in summary statistics between groups, we used Mann–Whitney rank-sum and chi2 tests, respectively.

We performed subgroup analyses in patients with T2D and in the control group. For each group, we performed univariate logistic regression with adjustment for age, male sex, BMI, CRP, dyslipidemia, adipose tissues (CATi, VATi, SATi), severe lung lesions (> 50%), leukocytes, eGFR, C-reactive-protein, Troponin-T and glycemia to analyze independent relationships of adipose tissue parameters and the primary endpoint (composite outcome of ICU or Death). Finally, we build different multivariate logistic regression models according to the clinical relevance, univariate statistical associations, and the absence of collinearity.

To assess the prognostic performance of the adipose tissue biomarkers (CATi/VATi/SATi) for the prediction of outcome (ICU admission or death), ROC curves were generated for each subgroup. We further determined the optimal threshold values for maximization of the sensitivity and specificity according to Youden’s index. P-values  < 0.05 were considered statistically significant. All statistical analysis were performed using JMP Pro version 16 (SAS Institute Inc., Cary, NC, USA, 1989–2022) and graphs were created with GraphPad Graphics software (San Diego, California USA).

## Results

Two hundred and two patients were included in this study. Baseline characteristics are described in Table 1. Patients with T2D had a higher BMI (27 vs 24 kg/m^2^, p = 0.0003), more hypertension (76% vs. 51%, p = 0.0003) and dyslipidemia (57% vs. 31%, p = 0.0001) than patients in the control group. Adipose tissue imaging biomarkers were significantly higher in the T2D group including CATi (124 vs 97 mL/m^2^, p = 0.02), VATi (709 vs 440 mL/m^2^, p < 0.0001) and SATi (513 vs 368 mL/m^2^, p < 0.01). We found also more severe coronary artery calcification (CAC-DRS = 3) in patients with T2D (24% vs 10%, p = 0.008). The CT lung severity score was not different between groups. Outcomes were similar across groups. At day 21 after admission, 42 patients (21%) had died from COVID-19, 48 (24%) required intensive care and 112 (55%) were admitted to in a conventional care unit (CMU).

**Table 1:** Baseline characteristics of the included population and comparison between diabetes and controls

Parameters	Overall N = 202	Control N = 108	Diabetes N = 94	P
Anthropometry				
Age, years (IQR)	71 (59–81)	76 (59–83)	70 (61–77)	0.07
Male, n (%)	127 (63)	74 (61)	72 (70)	0.06
Weight (kg)	73 (64–82)	70 (61–79)	78 (67–88)	**0.0003**
BMI (kg/m^2^)	25 (23–29)	24 (22–27)	27 (24–31)	**0.0003**
Cardiometabolic risk profile				
Active smoking, n (%)	14 (7%)	8 (7)	6 (7)	1.00
Hypertension, n (%)	126 (62%)	55 (51)	71 (76)	**0.0003**
Dyslipidemia, n (%)	87 (43%)	33 (31)	54 (57)	**0.0001**
CVDs, n(%)	49 (24)	14 (13)	35 (37)	** < 0.0001**
Adipose tissue imaging biomarkers				
CATi (mL/m^2^)	113 (69–152)	97 (54–150)	124 (85–155)	**0.0160**
VATi (mL/m^2^)	545 (299–852)	440 (231–645)	709 (416–999)	** < 0.0001**
SATi (mL/m^2^)	443 (268–634)	368 (239–562)	513(3 39–710)	** < 0.01**
VATi/CATi	5.5 (3.4–7.7)	5 (2.8–6.7)	5.9 (4–8.4)	** < 0.01**
CAC-DRS classification				
0	46 (23)	28 (26)	18 (19)	0.03
1	81 (40)	43 (40)	38 (40)	
2	41 (20)	26 (24)	15 (16)	
3	34 (17)	11 (10)	23 (24)	
> 0	46 (23)	28 (26)	18 (19)	0.31
3 vs < 3	34 (17)	11 (10)	23 (24)	**0.0082**
Lung CT scan severity score				
Minimal (10%)	32 (16)	14 (13)	18 (19)	0.06
Moderate (10–25%)	67 (33)	37 (34)	30 (32)	
Extensive (25–50%)	74 (37)	41 (38)	33 (35)	
Severe (> 50%)	29 (14)	16 (15)	13 (14)	
Minimal (10%) vs > 10%	32 (16)	14 (13)	18 (19)	0.70
Severe (> 50%) vs ≤ 50%	29 (14)	16 (15)	13 (14)	1.00
Biological markers				
Leukocytes (× 10^9^/L)	6.4 (4.9–8.4)	6.1 (4.4–7.9)	6.5 (5.3–8.6)	0.05
PMNs (× 10^9^/L)	4.9 (3.4–6.7)	4.6 (3.1–6.2)	5.2 (3.7–7)	0.07
Lymphocytes (× 10^9^/L)	0.9 (0.7–1.2)	0.8 (0.7–1.1)	1 (0.7–1.3)	**0.02**
CRP (mg/L)	70 (30–131)	67 (29–134)	79 (34–129)	0.65
Platelets (× 10^3^/L)	212 (155–267)	204 (146–269)	215 (166–267)	0.31
Troponin-T (ng/L)	18 (9–38)	15 (8–30)	20 (11–45)	**0.01**
AST (UI/L)	44 (31–64)	45 (33–62)	44 (29–67)	0.61
ALT (UI/L)	31 (20–49)	31 (21–49)	32 (20–52)	0.67
eGFR(mL/min/1.73 m^2^)	85 (60–110)	88 (66–123)	76 (48–100)	**0.009**
Glycemia (mmoL/L)	6.9 (5.5–9.4)	5.6 (5.1–6.6)	9.4 (7–11.8)	** < 0.0001**
Outcomes				
CMU, n (%)	112 (55)	58 (54)	54 (57)	0.74
ICU, n (%)	48 (24)	28 (26)	20 (21)	
Death, n (%)	42 (21)	22 (20)	20 (21)	
ICU admission or Death, n (%)	90 (45)	50 (46)	40 (43)	0.67
Mortality, n (%)	42 (21)	22 (20)	20 (21)	1.00

Data are represented as median (interquartile range) and frequency (percent) as appropriate. Median and frequency differences were tested using Mann–Whitney and chi2 tests. respectively

*BMI* body mass index, *CVDs* cardiovascular disease, *CRP* C-reactive protein, *CMU* conventional medical care unit, *ICU* intensive care unit, *CATi* cardiac adipose tissue index, *VATi* visceral adipose tissue index, *SATi* subcutaneous adipose tissue index, *CAC-DRS* coronary artery calcium data and reporting system, *PMNs* polymorphonuclear neutrophils. Note: P values in bold indicate statistical significance P<0.05

### Value of clinical, biological and imaging parameters for the prediction of early ICU admission or death in the overall population

**Fig. 2:**
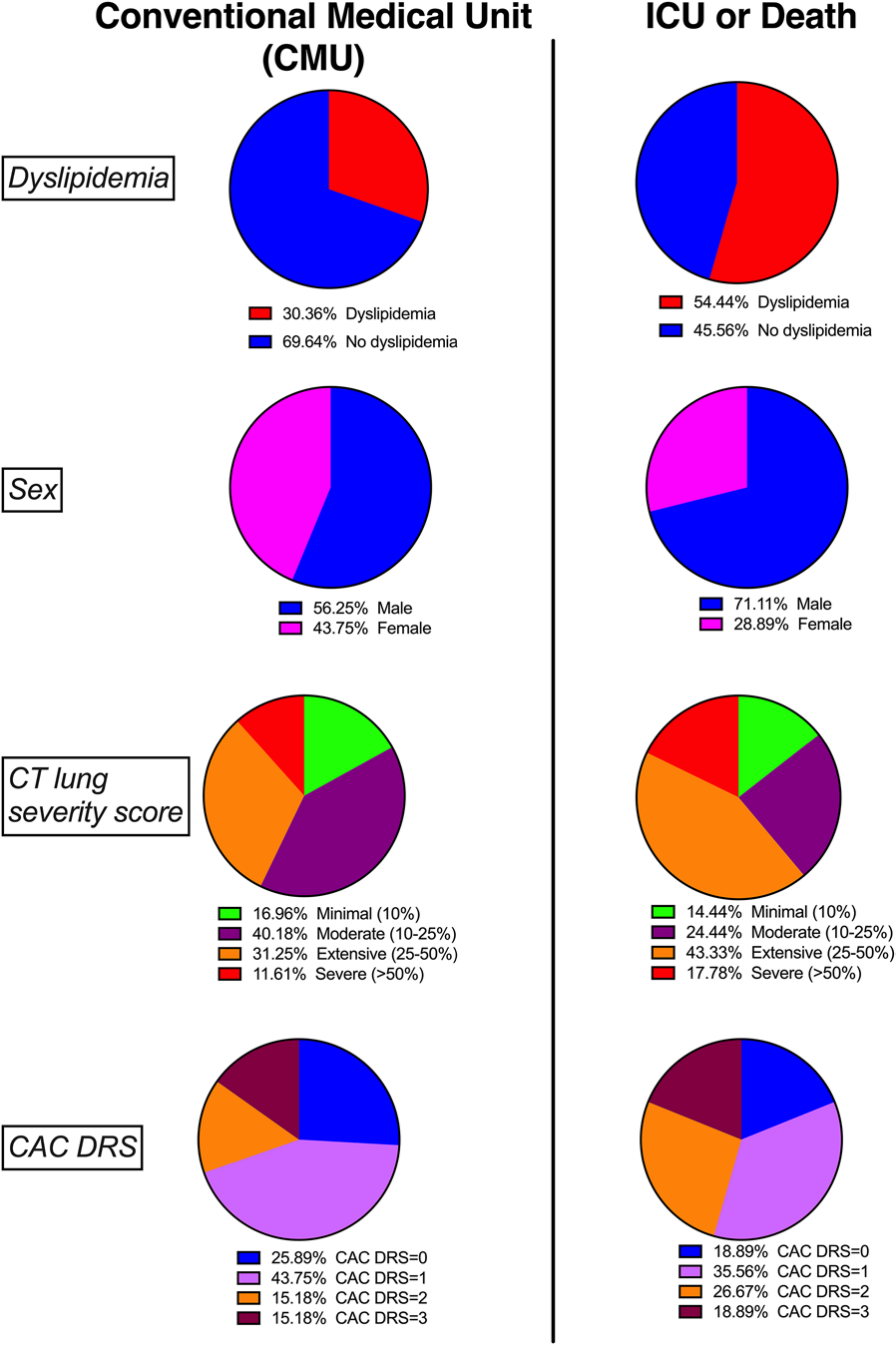
Determinants of severe short-term outcome in the overall study population

The distributions of clinical, biological and imaging parameters according to the outcome are summarized in Table 2 and Fig. 2. In the overall study population, male sex (71 vs 56%, p = 0.04), dyslipidemia (54 vs 34%, p = 0.003), hyperleukocytosis (6.8 vs 6, p = 0.005), elevated CRP (93 vs 60, p = 0.03) and increased T-troponin level (23 vs 15, p = 0.0007) were associated with early ICU admission or death. Adipose tissue biomarkers were not statistically different between outcome categories when considering the overall population. The lung CT severity score was also not different between outcome categories.

**Table 2:** Distributions of clinical, biological and imaging parameters according to the outcome

	CMU N = 112	ICU OR death N = 90	P-value
Anthropometry			
Age, years (IQR)	71 (56–81)	71 (63–80)	0.31
Male, n (%)	63 (56)	64 (71)	**0.04**
BMI (kg/m^2^)	25 (22–29)	26 (23–29)	0.63
Cardiometabolic risk profile			
Active smoking, n (%)	7 (6)	7 (8)	0.78
Hypertension, n (%)	67 (60)	59 (66)	0.47
Dyslipidemia, n (%)	38 (34)	49 (54)	**0.003**
CVDs, n (%)	25 (22)	24 (27)	0.51
Adipose tissue imaging biomarkers			
CATi (mL/m^2^)	102 (66–145)	116 (70–157)	0.12
VATi (mL/m^2^)	506 (291–801)	605 (357–931)	0.26
SATi (mL/m^2^)	423 (299–640)	452 (255–621)	0.79
CATi/VATi	0.17 (0.12–0.25)	0.18 (0.13–0.24)	0.76
Lung CT severity score			
Minimal (10%)	19 (17)	13 (14)	0.06
Moderate (10–25%)	45 (40)	22 (24)	
Extensive (25–50%)	35 (31)	39 (43)	
Severe (> 50%)	13 (12)	16 (18)	0.23
Minimal (10%) vs > 10%	19 (17)	13 (14)	0.70
Severe (> 50%) vs ≤ 50%	13 (12)	16 (18)	0.23
CAC-DRS Classification			
0	29 (26)	17 (19)	0.14
1	49 (44)	32 (36)	
2	17 (15)	24 (27)	
3	17 (15)	17 (19)	
> 0	29 (26)	17 (19)	0.31
3 vs < 3	17 (15)	17 (19)	0.57
Biological markers			
Leukocytes (× 10^9^/L)	6 (4.4–7.3)	6.8 (5.6–8.9)	**0.005**
Lymphocytes (× 10^9^/L)	1 (0.7–1.3)	0.8 (0.7–1.2)	0.08
Platelets (× 10^9^/L)	215 (160–261)	206 (146–273)	0.38
eGFR (mL/min/1.73 m^2^)	86 (61–116)	80 (59–100)	0.46
CRP (mg/L)	60 (29–105)	93 (31–143)	**0.03**
Troponin-T (ng/L)	15 (8–30)	23 (12–46)	**0.0007**

Data are represented as median (interquartile range) and frequency (percent) as appropriate. Median and frequency differences were tested using Mann–Whitney and chi2 tests. respectively

*BMI* body mass index, *CRP* C-reactive protein, *CMU* conventional medicale care unit, *ICU* intensive care unit, *CATi* cardiac adipose tissue index, *VATi* visceral adipose tissue index, *SATi* subcutaneous adipose tissue index, *CAC-DRS* coronary artery calcium data and reporting system. Note: P values in bold indicate statistical significance P<0.05

### Value of clinical, biological and imaging parameters for the prediction of early ICU admission or death in T2D and controls

Univariate and multivariate analysis of the main clinical, biological and radiological findings are detailed in Table 3, 4 respectively.

**Table 3:** Univariate analysis of variables for the prediction of ICU or death in the overall population and subgroups

	Overall (n = 202)			Controls (n = 108)			T2D(n = 94)		
	OR	95% CI	p	OR	95% CI	p	OR	95% CI	p
Age, years	1.01	1.00–1.04	0.14	1.03	1.00–1.05	**0.04**	0.99	0.96–1.03	0.67
Male sex	1.91	1.06–3.45	**0.03**	1.78	0.83–3.90	0.14	2.35	0.93–6.38	0.07
BMI, kg/m^2^	1.01	0.95–1.07	0.78	0.97	0.89–1.04	0.40	1.08	0.99–1.19	0.09
Dyslipidemia	2.33	1.32–4.12	**0.003**	1.93	0.85–4.48	0.12	3.75	1.57–9.50	**0.003**
CATi, mL/m^2^ (continuous)	1.005	1.0003–1.01	**0.03**	1.00	0.99–1.01	0.99	1.01	1.00–1.02	**0.001**
CATi > 100 mL/m^2^ (dichotomous)	1.52	0.87–2.67	0.15	0.73	0.34–1.57	0.42	4.71	1.78–12.49	**0.002**
VATi, mL/m^2^	1.00	1.00–1.00	0.24	1.00	1.00–1.00	0.94	1.001	1.00–1.002	**0.046**
SATi, mL/m^2^	1.00	1.00–1.00	0.64	1.00	1.00–1.00	0.30	1.00	1.00–1.00	0.61
Severe lung lesions (> 50%)	1.64	0.75–3.69	0.22	1.17	0.38–3.81	0.78	1.21	0.43–3.59	0.73
CAC DRS = 3	1.30	0.62–2.74	0.48	3.49	0.9–14	0.06	0.83	0.32–2.17	0.70
Leukocytes, × 10^9^/L	1.08	0.99–1.18	0.09	1.11	0.98–1.28	0.09	1.05	0.94–1.19	0.38
eGFR, mL/min/1.73 m^2^	0.99	0.99–1.00	0.14	0.99	0.98–1.00	0.23	0.99	0.98–1.00	0.26
CRP, mg/L	1.005	1.001–1.008	**0.007**	1.00	1.00–1.01	0.06	1.01	1.00–1.01	**0.045**
Troponin-T, ng/L	1.00	1.00–1.01	0.10	1.01	1.00–1.02	0.09	1.00	1.00–1.01	0.42
Glycemia, mL	1.05	0.96–1.14	0.27	1.29	0.93–1.82	0.12	1.07	0.96–1.21	0.22

*BMI* body max index, *CATi* cardiac adipose tissue index, *VATi* visceral adipose tissue index, *SATi* subcutaneous adipose tissue index, *CAC-DRS* coronary artery calcium data and reporting system. Note: P values in bold indicate statistical significance P<0.05

**Table 4:** Multivariate models for the prediction of death or ICU admission in T2D and control patients

	Controls			T2D		
	OR	95% CI	p	OR	95% CI	p
MODEL 1						
Age, years	1.04	1.01–1.07	**0.02**	1	0.94–1.02	0.29
Male sex	2.4	1.01–5.7	**0.04**	1.7	0.6–4.7	0.34
BMI, kg/m^2^	1	0.93–1.11	0.67	1.1	0.96–1.17	0.25
CATi, mL/m^2^ (continuous)	1	0.99–1.004	0.34	1.01	1.003–1.02	**0.005**
MODEL 2						
CATi, mL/m^2^ (continuous)	1	0.99–1.01	0.6	1.01	1.003–1.02	**0.005**
Dyslipidemia	1.7	0.7–4.3	0.3	3.7	1.4–10.2	**0.008**
CRP, mg/L	1	0.999–1.001	0.09	1	0.998–1.01	0.1
CAC DRS = 3 or < 3	2.5	0.6–11	0.2	0.5	0.2–1.5	0.2
MODEL 3						
CATi > 100 mL/m^2^ (dichotomous)	0.6	0.3–1.4	0.3	4.6	1.6–14	**0.004**
Dyslipidemia	1.8	0.7–4.5	0.2	3.8	1.4–10	**0.007**
CRP, mg/L	1	0.999–1.01	0.1	1	0.998–1.01	0.1
CAC DRS = 3 or < 3	2.5	0.6–12	0.2	0.5	0.2–1.5	0.2

*CATi* cardiac adipose tissue index. *BMI* Body mass index, *CRP* C reactive protein, *CAC-DRS* coronary artery calcium and data system. Note: P values in bold indicate statistical significance P<0.05

Model 1: Age, Male Sex, BMI, CATi. Controls: p = 0.07. Diabetes: p = 0.005

Model 2: Dyslipidemia, CRP,CAC = 3, CATi. Controls: p = 0.11. Diabetes: p = 0.0002

Model 3: Dyslipidemia, CRP,CAC = 3, CATi > 100 mL/m^2^. Controls: p = 0.07. Diabetes: p = 0.0001

In the T2D group (n = 94), CATi as a continuous variable (p = 0.001) or as a dichotomous variable (OR = 4.71, p = 0.002), dyslipidemia (OR = 3.75, p = 0.003) and VATi (p = 0.046) were related to ICU admission or death in univariate analysis (Table 3). After adjustment for age, sex and BMI in model 1 and for dyslipidemia, CRP, CAC-DRS = 3 (severe coronary artery calcifications) in model 2, CATi remained independently and significantly associated with ICU admission or death (p = 0.005) (Table 4).

In the control group (n = 108), only advanced age was associated with ICU admission or death in univariate analysis (p = 0.04) whereas dyslipidemia, biological markers and imaging biomarkers were not associated with ICU admission or death. In the multivariate analysis, dyslipidemia and CATi were not associated with death or ICU admission in controls (Table 4).

### Comparison of cardiac, subcutaneous and visceral abdominal adipose tissue depots with respect to outcome

**Fig. 3:**
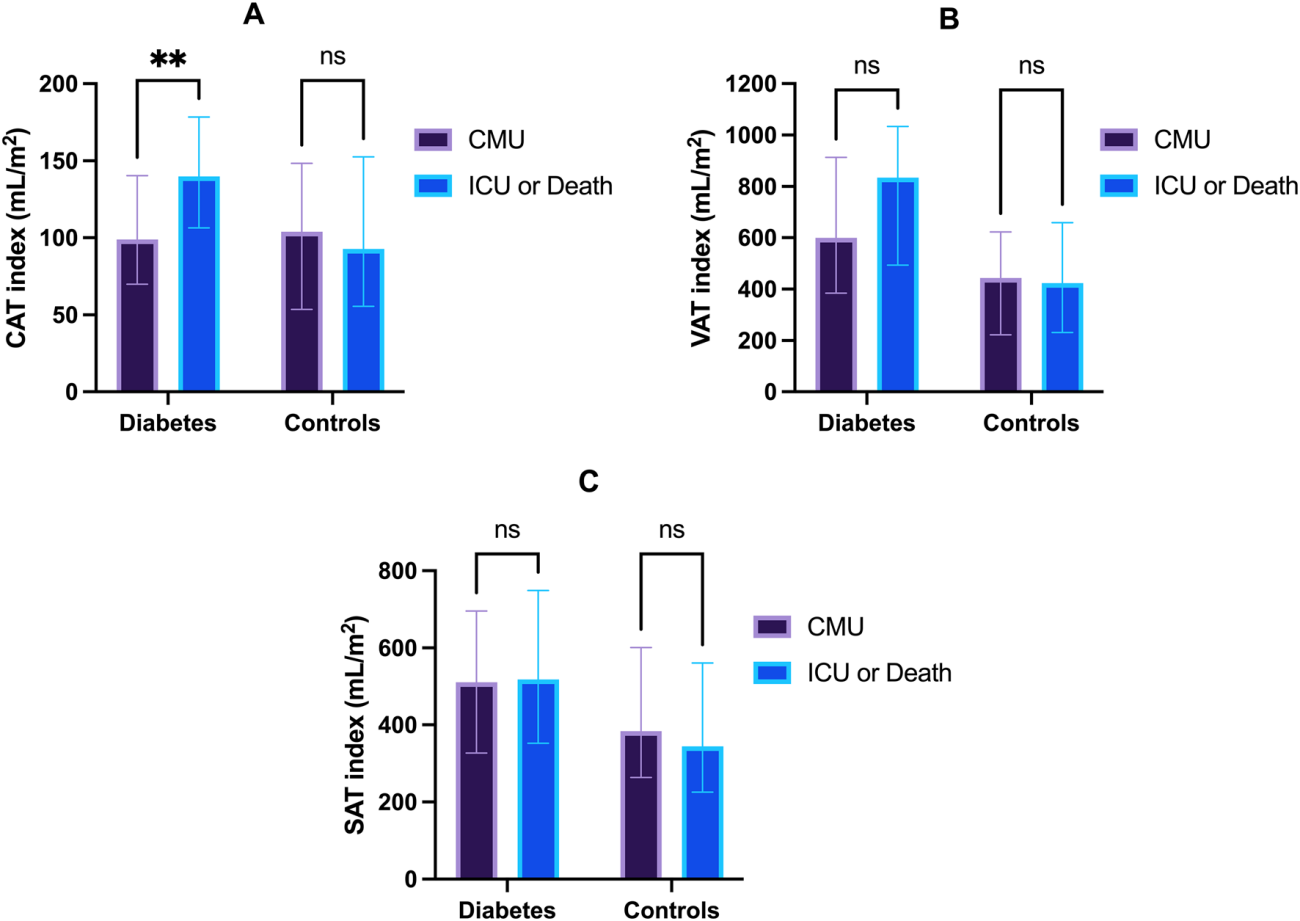
Comparison of CATi (**A**), VATi (**B**), SATi (**C**) according to the short-term outcomes for diabetes and controls patients. Data are represented as median with interquartile range. *CMU* conventional medical care unit, *ICU* intensive care unit, *CAT* cardiac adipose tissue, *VATI* visceral adipose tissue, *SAT* subcutaneous adipose tissue: non significative, **p < 0.005

In the T2D group, CATi (140 vs 99 mL/m^2^, p = 0.004) was significantly increased for patients admitted in ICU or deceased compared with those admitted in CMU whereas VATi and SATi were not significantly different (Fig. 3). In controls, adipose tissue biomarkers (CATi, VATi and SATi) were not statistically different between patients admitted in CMU and those with a worse prognosis (ICU admission or death) (Fig. 3).

**Fig. 4:**
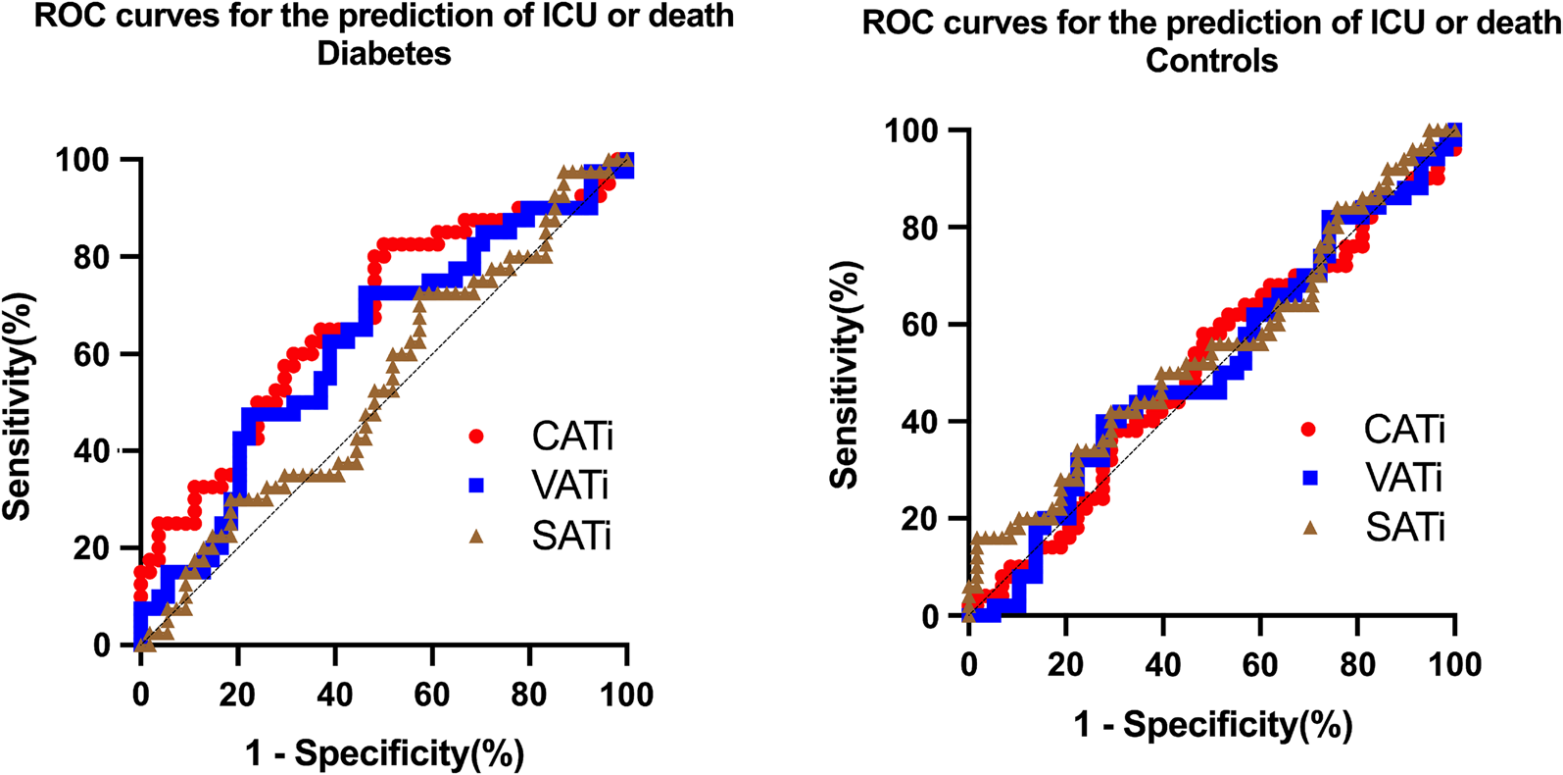
Receiver operating curves (ROC) of the different fat depots (CATi, VATi and SATi) for the prediction of ICU or Death in T2D and control patients. For T2D patients, diagnostic performance of CATi (AUC = 0.67, p = 0.0011) and VATi (AUC = 0.62, p = 0.051) was higher compared to SATi (AUC = 0.54, p = 0.57). Adipose tissue imaging biomarkers were not statistically significant for the prediction of ICU or death in non-diabetic patients. *AUC* area under the curve *CATi* cardiac adipose tissue index. *VATi* visceral adipose tissue index. *SATi* subcutaneous adipose tissue index

In the T2D group, AUC of CATi was 0.67 (p = 0.004) with 100 mL/m^2^ as an optimal cut-off value reaching a sensitivity of 83% and a specificity of 50% for the prediction of early ICU admission or death (Fig. 4). In multivariate analysis, an increased CATi > 100 mL/m^2^ was strongly related to ICU admission or death (OR = 4.6, p = 0.004, Table 4) even after adjustment for dyslipidemia, CRP and CAC-DRS = 3. For VATi and SATi, AUC were 0.62 (p = 0.051) and 0.54 (p = 0.57) respectively (Fig. 3). In the control group, AUCs for the three adipose tissue depots were low and non significantly related to ICU or death (Fig. 4).

## Discussion

Our study found that, in COVID-19, cardiac adipose tissue index (CATi) measured by CT is an independent predictor of severe short-term outcome specific to T2D patients when compared to non-diabetics. Furthermore, the relationship to adverse short-term events was stronger for CATi compared to abdominal visceral and subcutaneous adipose tissue even after indexation to body surface area.

### Overall study population

The characteristics of our cohort were similar to other published cohorts [3, 13, 14] in COVID-19 in the distribution of age and sex ratio. However, lung involvement was somewhat less severe in our study population. Compared to the study by Bihan et al. [11] patients in our study were more frequently diabetic by design but also less obese and had an overall worse prognosis. Indeed, three weeks after admission, among the 202 patients of the present cohort, 90 (45%) presented critical clinical evolution (intensive care requirement or death) while 112 (55%) remained hospitalized in a conventional medical unit. Male gender, dyslipidemia and CRP were predictors of early ICU or death in the overall population. This confirms the potential contribution of dysmetabolism-related low-grade inflammation as key factors for adverse prognosis in the overall cohort (Fig. 2).

### Diabetic patients with COVID-19 have a specific risk profile partly mediated by CAT

Markers of dysmetabolism such as dyslipidemia and increased epicardial and visceral fat seem to play a determinant role in indicating individual risk in diabetics with COVID-19. In patients with insulin resistance, prediabetes or T2D, excess of cardiac adipose tissue is generally associated with increased visceral adipose tissue and stable or reduced subcutaneous abdominal adipose tissues. We previously reported [8] that increased CATi and circulating IL-6 levels relate significantly to early mortality and ICU requirement during COVID-19 infection in patients with type 2 diabetes. In the present study, we evaluated the predictive values of three troncular fat depots (cardiac, visceral and subcutaneous adipose tissues) for early adverse outcome including death and ICU in consecutive COVID-19 patients with or without diabetes. Of note, CATi remained a significant determinant of adverse outcome after adjustment for previously described risk factors in the COVID-19 [8] setting such as age, sex and BMI suggesting relative independence from obesity and aging processes. Together with increased CATi, dyslipidemia and increased CRP were also associated to unfavorable outcome in diabetic patients suggesting a conundrum of metabolic and inflammatory pathways playing a central role in the severity of COVID-19 in diabetic patients. Furthermore, coronary artery calcification was not associated with ICU admission or death. These results suggest the relative specificity of the cardiac and visceral fat components for diabetic patients which is higher in this group than in the control group and associated with outcome which may result from direct myocardial injury and not necessarily mediated by acute ischemic coronary events [15]. This is consistent with a recent meta-analysis [16] which demonstrated the pro-inflammatory role of visceral fat more elevated in diabetic patients and the association between IL6 and CATi. Both diabetes-related increase in cytokines and immune mediated response and direct viral injury on the myocardium via glycated ACE2 receptors may be mechanisms involved as in COVID-19 [17, 18]. It is well accepted that increased CAT depots not only increase the risk but also worsen the cardiovascular prognosis of T2D patients independently of classical cardiovascular risk factors [19]. Beyond myocardial ischemia, development of CAT as a source of low-grade inflammation seems a better predictor of adverse outcome than glucose level or HbA1c as previously discussed [9]. Failure of lipogenesis, increased lipolysis and inflammation are changes observed during CAT expansion [20]. Increased CAT secretes various inflammatory cytokines (among them IL-6, TNF-α, MCP-1) that contribute to local and systemic inflammatory environment [21]. COVID-19 infection is known to induce major systemic inflammation that can lead to severe complications and death [22]. Thus, by various mechanisms, the observation that type 2 diabetes patients are at high risk of severe outcome during COVID-19 can be explained by an exacerbation of the chronic low-grade inflammation in this population. In line with this assumption, ectopic fat depots and generation of low-grade inflammation are directly involved in multi-organ failure and death.

### Comparison of diabetic and non-diabetic individuals

Bihan et al. showed a relationship between increased CAT volume and disease aggravation in a global COVID-19 population sample including a subset of diabetic patients [11]*.* Since we previously published that CATi [8] was a strong predictor of adverse outcome in T2D patients, we next analyzed if this finding is also applicable to normoglycemic individuals. Diabetic and non-diabetic groups were comparable for their age, gender, red and white cell counts, platelets, liver function and lung scan severity score. As expected, patients with T2D differed from controls by increased body weight, BMI, hypertension, dyslipidemia and history of cardiovascular disease. All adipose tissue imaging parameters (CATi, VATi, SATi) were significantly higher in patients with T2D confirming that higher BMI in diabetic patients developed through excess of troncular adiposity. We showed that CATi (continuous or dichotomous CATi > 100 ml/m^2^) and dyslipidemia are associated with ICU admission or death in diabetic patients while only age could predict adverse outcome in controls. This emphasizes the deleterious properties of CATi in the context of COVID-19 infection in patients with T2D.

Our results are consistent with existing knowledge on CAT physiopathology. Indeed, CATi remained independently associated with a worse prognosis in diabetic patients after multiple adjustments (age, male sex, BMI for model 1; or dyslipidemia, CRP and CAC = 3 for model 2). Importantly, even if CATi index overlapped between both groups, this parameter cannot explain the clinical evolution of controls during COVID-19 infection since CATi and related inflammation variables are not significantly associated with outcome in controls. This important finding suggested that increased CAT volume may not be sufficient by itself to explain secretion of inflammatory cytokines by this tissue but needed activation of other key mechanisms to induce local low-grade inflammation. Among them, local development of hypoxia (as observed in other fat depots) induced local increasing numbers of macrophages and T lymphocytes and shift their metabolic profile toward inflammatory immune cells [23]. Controls and diabetic patients may markedly differ in such metabolism-related processes and this may explain why CATi did not relate to clinical outcome in controls. In this group, we identified age as the only one predictor of adverse outcome. This variable is a classical risk factor of death associated with COVID-19 infection. Intriguingly, if controls were characterized by a lower amount of CAC than diabetic patients, we described a non-significative trend association between severe CAC and early adverse outcomes in the control group. Such distribution may also be a consequence of age in controls and of the combination of age and inflammation in patients with T2D. Thus, diabetic patients and controls differ by their respective pathophysiology during COVID-19 infection, driven by excess of truncular adiposity and inflammation for the former and classical aging and atherosclerosis (CAC) mechanisms for the latter.

### Study limitations

There are some limitations to this study. First of all, it is a single-center study. Secondly, the results need to be further verified by large prospective studies. Thirdly, in contrast to diabetic patients, IL-6 levels were not assessed in controls during the first lockdown. As a consequence, we chose CRP as a biomarker of systemic inflammation. CRP is produced following the increased synthesis of proinflammatory cytokines and is a acute phase protein in the innate immune response [24]. Even if we demonstrated that IL-6 is an important member of the cytokine network during the COVID-19 infection, serum CRP levels has often been used as a marker of inflammation. Adverse prognosis of high CRP levels following COVID-19 infection has been demonstrated such as positive correlation between IL-6 and CRP levels or the use of CRP and IL-6 levels to predict severe and fatal COVID-19 infection [25].

Technical issues remain concerning the absence of standardization of CAT measurement with CT despite an intrinsic superiority to echocardiography as it allows CAT volumetry CAT and the recent availability of semi-automated segmentation methods. In particular the value of CAT attenuation remains to be established as it has been shown to be highly heterogeneous [10] and potentially influenced by corticosteroid [26], statin or colchicine therapy [27]. Nevertheless, taken together, our data emphasize CATi index as one of the most important and specific predictors of outcome during COVID-19 infection in diabetic patients. Indeed, these parameters in contrast failed to predict outcome in controls despite some overlap in adipose tissue imaging biomarkers and CRP levels in both groups.

## Conclusions

Our data showed the specificity of cardiac adipose tissue assessed using CT in diabetic patients to predict early ICU admission or death in consecutive patients hospitalized for COVID-19. This automated non-invasive CT measurement could be used in daily practice to optimize the management of T2D patients with COVID-19 infection.

## Data Availability

The datasets generated during and/or analyzed during the current study are not publicly available due to storage under the umbrella of the Paris Academic Hospitals Group Assistance Publique-Hôpitaux de Paris EDS research data storage warehouse but may be available from the corresponding institution on reasonable request.
